# Enhanced phosphatidylserine-selective cancer therapy with irradiation and SapC-DOPS nanovesicles

**DOI:** 10.18632/oncotarget.26615

**Published:** 2019-01-25

**Authors:** Harold W. Davis, Subrahmanya D. Vallabhapurapu, Zhengtao Chu, Swarajya L. Vallabhapurapu, Robert S. Franco, Michelle Mierzwa, William Kassing, William L. Barrett, Xiaoyang Qi

**Affiliations:** ^1^ Division of Hematology/Oncology, Translational Research Laboratory, Department of Internal Medicine, University of Cincinnati College of Medicine, Cincinnati, OH, USA; ^2^ Department of Radiation Oncology, University of Cincinnati College of Medicine, Cincinnati, OH, USA; ^3^ Division of Human Genetics, Department of Pediatrics, Cincinnati Children's Hospital Medical Center, Cincinnati, OH, USA

**Keywords:** radiotherapy-induced resistance, surface phosphatidylserine-selective, cancer cell death, SapC-DOPS nanovesicles, combined-treatment enhancement

## Abstract

Normal living cells exhibit phosphatidylserine (PS) primarily within the intracellular leaflet of the plasma membrane. In contrast, viable cancer cells have high levels of PS on the external surface, and exhibit a broad range of surface PS, even within specific types of cancer. Agents that target surface PS have recently been developed to treat tumors and are expected to be more effective with higher surface PS levels. In this context, we examined whether surface PS is increased with irradiation. *In vitro* irradiation of cancer cell lines selected surviving cells that had higher surface PS in a dose- and time-dependent manner. This was more pronounced if surface PS was initially in the lower range for cancer cells. Radiation also increased the surface PS of tumor cells in subcutaneous xenografts in nude mice. We found an inverse relationship between steady state surface PS level of cancer cell lines and their sensitivity to radiation-induced cell death. In addition, serial irradiation, which selected surviving cells with higher surface PS, also increased resistance to radiation and to some chemotherapeutic drugs, suggesting a PS-dependent mechanism for development of resistance to therapy. On the other hand, fractionated radiation enhanced the effect of a novel anti-cancer, PS-targeting drug, SapC-DOPS, in some cancer cell lines. Our data suggest that we can group cancer cells into cells with low surface PS, which are sensitive to radiation, and high surface PS, which are sensitive to SapC-DOPS. Combination of these interventions may provide a potential new combination therapy.

## INTRODUCTION

Phospholipids are arranged asymmetrically in cell membranes with neutral phospholipids on the outer leaflet and anionic phospholipids [1] located primarily on the inside of the membrane [1–3]. An early event in apoptosis is the appearance of phosphatidylserine (PS) on the surface of the cell. This PS alerts phagocytic cells to engulf the cell and thus reduces the inflammatory response [4, 5]. Elevated external PS is also found in non-apoptotic primary and metastatic cancer cells, and in their associated tumor vasculature [3, 6–8]. Furthermore, viable cancer cells with high external PS are resistant to phagocyte-mediated removal. Macrophages recognize PS on the surface of apoptotic cells [9] but cancer cells appear to repel macrophages by displaying CD47, which inhibits phagocytosis [10]. Recent studies have indicated that this surface PS may be exploited as a significant target for cancer therapy [3, 7, 8]. Indeed, the PS-targeting antibody, bavituximab [11], and the PS-binding peptide-peptoid hybrid, PPS1D1 [12], have been used successfully to trigger cytotoxicity of tumor-associated endothelial cells or cancer cells both *in vitro* and *in vivo*.

The loss of PS asymmetry in cancer cells may be due to reduced activity of ATP-dependent phospholipid translocases (flippases) and/or elevated activity of phospholipid scramblase, perhaps related to high levels of intracellular calcium (Ca^2+^_i_) [13, 14]. While cancer cells generally have higher amounts of PS on the surface, the amount varies greatly among cultured cell lines, even within the same class of cancer [6, 15]. We have recently demonstrated that cancer cells with high external PS have reduced flippase activity and high Ca^2+^_i_ compared to cancer cells with lower surface PS [14]. The high surface PS cells also have elevated total cellular PS [14]. Importantly, this increased PS is inducible and not associated with programmed cell death [8, 16].

Radiotherapy is a common treatment for malignant tumors and has been shown to improve outcomes when used with chemotherapy [17, 18]. However, radiation can damage off target tissues and there is evidence that it can promote tumor growth by stimulating angiogenesis and cancer cell migration [19, 20]. Interestingly, radiation increases surface PS on tumor blood vessels and this has been used successfully employed in mouse models of lung cancer and glioblastomas with the PS-targeting antibody, bavituximab [21–23]. While this work was aimed at the surface PS on endothelial cells we decided to examine the effects of radiation on tumor cell PS. SapC-DOPS, an anti-cancer protein/lipid nanovesicle developed in our laboratory targets surface PS and kills cancer cells both *in vitro* and *in vivo* [6, 11, 24, 25]. SapC-DOPS is composed of the natural lysosomal protein, Saposin C (SapC), and dioleoylphosphatidylserine (DOPS) [26, 27] and a Phase 1 clinical trial has just been completed showing that SapC-DOPS is very safe [28]. We investigated whether radiation could alter surface PS of cancer cells. Since SapC-DOPS performs better with high surface PS cells [6, 15, 29], we hypothesized that the high surface PS cells selected by irradiation may decrease the effects of subsequent irradiation or even chemotherapy but enhance susceptibility to SapC-DOPS treatment, thus introducing a potent new combination therapy.

## RESULTS

We examined the effects of single and serial dose irradiation on the surface PS of a number of cancer cells. In the clinic, fractionated radiation therapy is often used to protect the patients from a single high dose radiation exposure [30–32]. Therefore, we serially irradiated cells at 5 Gy once a week for several weeks to investigate whether this would alter surface PS or modify the effects we obtained with a single dose of radiation.

### A single dose of irradiation increases the surface PS of cancer cells *in vitro* and *in vivo*

**Figure 1:**
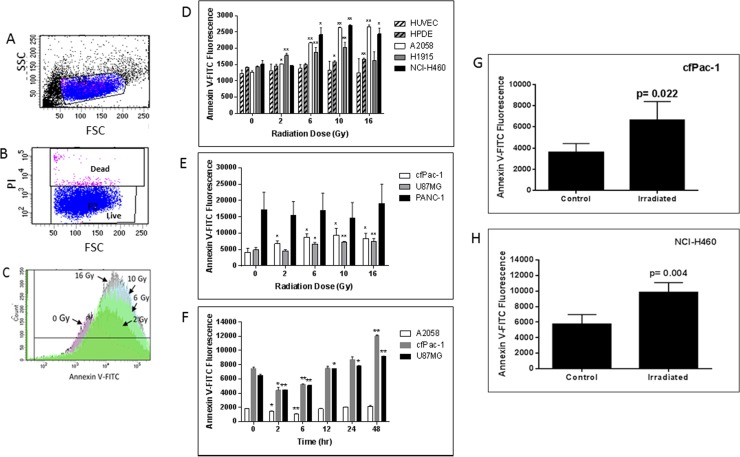
Radiation-induced increase in surface PS is dose- and time-dependent (**A**–**C**) cfPac-1 cells, a human pancreatic cancer cell line, were irradiated at 0, 2, 6, 10 and 16 Gy. 24 hr later Annexin V-FITC staining was performed and the cells were processed by flow cytometry. (**A**) Gating of cells. FSC: forward light scattering, an indication of cell volume; SSC: side light scattering, an indication of cellular granularity. (**B**) Gating of live cells. PI (propidium iodide) stains only dead cells. (**C**) Histogram of cells stained with Annexin V-FITC shows the effects of increasing doses of radiation on Annexin V staining (i.e. surface PS). (**D**–**H**) Cells were irradiated at the indicated doses and 24 hr later Annexin V-FITC staining was determined on low surface PS cells (less than 2000 fluorescent units; (**D**) or moderate or high surface PS cells (greater than 3000 fluorescent units; (**E**). (**F**) Cells were irratiated at 5 Gy and analyzed at the indicated times (A2058, cfPac-1 and U87MG). To examine whether tumor cell surface PS was also increased by radiation 2 × 10^6^ cfPac-1 (**G**) or NCI-H460 (**H**) cells were injected subcutaneously into nude mice. When the tumors were ~400 mm^3^ some were treated with 10 Gy of targeted irradiation as described in “Materials and Methods”. The tumors were removed 48 hr. later and the cells were dispersed into single cells. The cells in all panels were then processed and gated as in Figure 1. ^*^p < 0.05, ^**^p < 0.01. cfPac-1 and PANC-1 are pancreatic cancer cell lines; A2058 is a melanoma cell line; NCI-H460 and H1915 are metastatic lung cancer cell lines; U87MG is a glioblastoma cell line, HPDE is a normal, immortalized pancreatic cell line and HUVEC are primary human umbilical vein endothelial cells.

cfPac-1, a pancreatic cancer cell line with moderate surface PS, exhibited a radiation dose dependent increase in PS 24 hr after irradiation (Figure 1C). This increase in surface PS is on PI negative live cells as we detected very little cell death during the first 48 to 72 hours after the irradiation (even at 16 Gy). Although cell lines varied in their response, radiation increased overall surface PS populations in all of the cancer cells that we tested except for PANC-1, which has a very high initial level of surface PS (Figures 1D and 1E). In contrast, HUVEC (human umbilical vein endothelial cells) and HPDE cells (a normal pancreatic epithelial cell line) had little or no increase in surface PS (Figure 1D). For all cancer and normal cell lines the percentage of dead cells was less than 10% and did not change significantly in the first 48 hr following any of the radiation doses. The time dependency of PS change after irradiation was examined in several cancer cell lines, and an initial decrease was found before the increase (Figure 1F).

An increase in cell surface PS was also detected after irradiation of subcutaneous tumors formed after injection of cfPac-1 (Figure 1G) or NCI-H460 (Figure 1H). Although there were variable numbers of dead cells associated with the tumors, this did not change appreciably with irradiation. For cfPac-1 the percentage of dead cells was 1.1 ± 0.6 and 2.7 ± 0.8 for control and irradiated cells, respectively; for NCI-H460 it was 72.0 ± 15.0 and 65.9 ± 2.2. All of the PS data shown are on live (propidium iodide negative) cells.

### The increase in surface PS after a single irradiation is dependent on caspase activity

**Figure 2:**
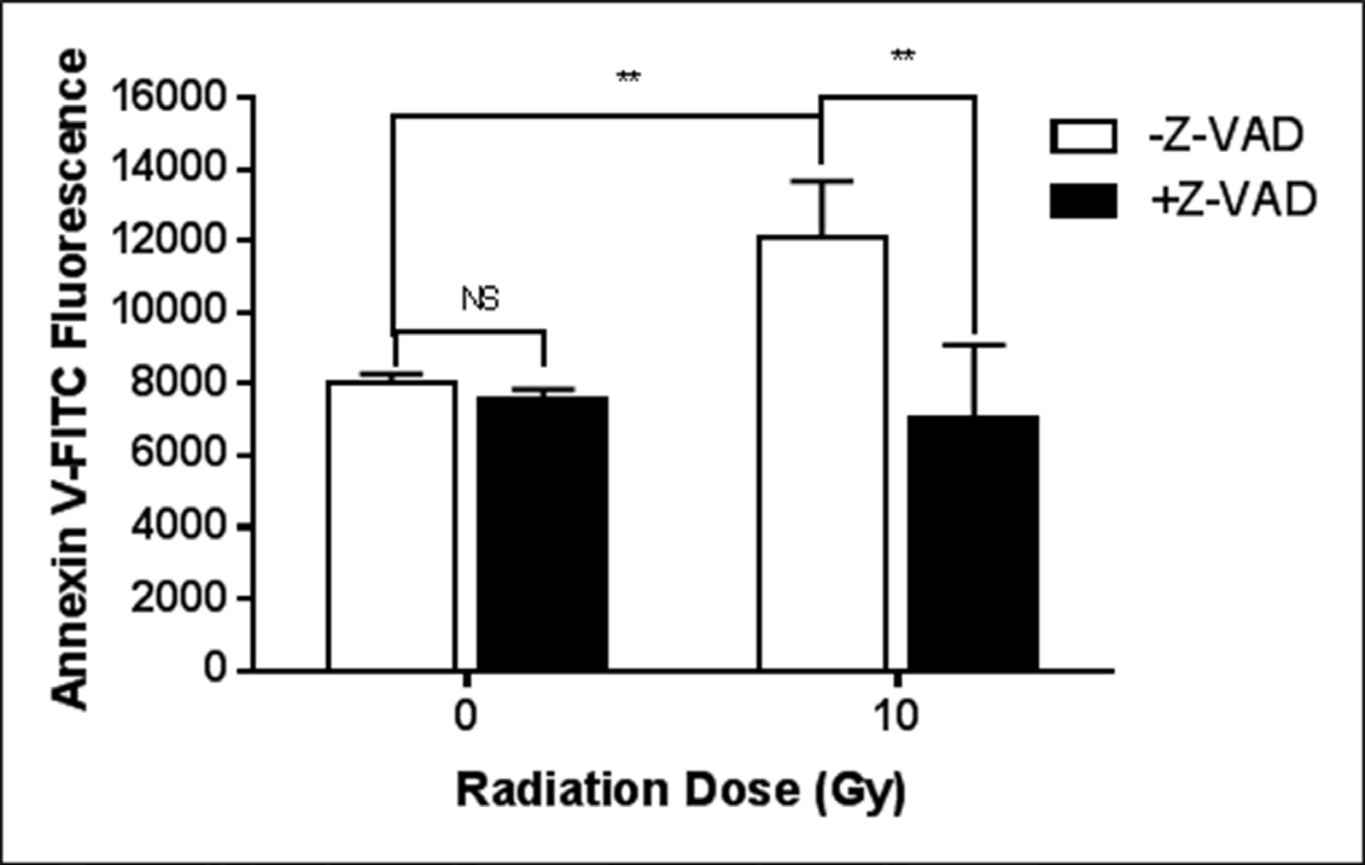
Caspase is critical for the radiation-induced exposure of PS cfPac-1 cells were irradiated at 10 Gy in the presence or absence of 10 μM Z-VAD-fmk, Sigma (St. Louis, MO, USA). 24 hr. later the cells were assessed for Annexin V binding as in Figure 1. ^**^p < 0.01, NS = not significantly different from control.

The pan-caspase inhibitor, Z-VAD fmk, completely eliminated the radiation-induced surface PS elevation (Figure 2). On the other hand, as shown in Table 1, the activities of flippase and scramblase are unchanged in cfPac-1 cells during the period when the cells are still responding to the 10 Gy irradiation by increasing surface PS. While there is a slight, insignificant decrease in scramblase activity, we would expect an increase in this activity if scramblases were involved in the radiation-induced increase in surface PS. Total PS and intracellular calcium were also unchanged (Table 1).

**Table 1:** The increase in surface PS caused by irradiation is unclear but does not appear to be due changes in intracellular calcium translocase activity or total PS

	Units	Control	Irradiated (10Gy)	
**Flippase**	Initial rate	2.03 ± 0.02	2.03 ± 0.07	NS
**Scramblase**	Initial rate	0.75 ± 0.19	0.33 ± 0.40	NS
**Total PS**	PS/PI ratio	7.50 ± 2.57	5.28 ± 1.95	NS
**Calcium**	arbitrary fluorescence	8717.3 ± 421.8	7245.5 ± 777.1	NS

The activities of the PS translocases, flippase and scramblase, the total amount of PS and intracellular calcium were determined in cfPac-1 cells 24 hr. after 10 Gy irradiation as described in “Materials and Methods”.

### Cancer cell surface PS is correlated with sensitivity to a single dose of irradiation

**Figure 3:**
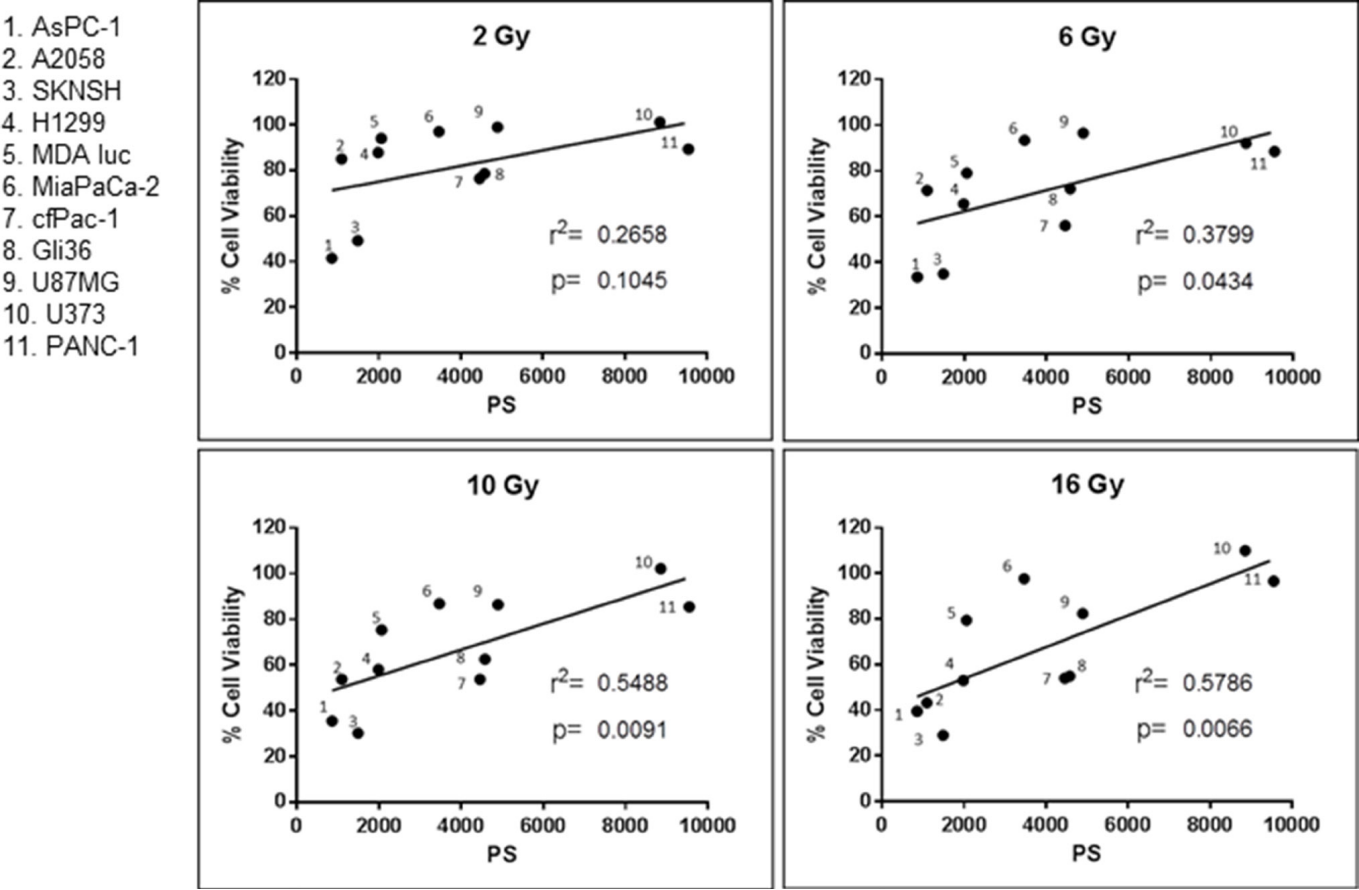
Correlation between cancer cell surface phosphatidylserine (PS) and resistance to radiation Surface PS from untreated cells was determined with Annexin V-FITC staining and cell viability was ascertained with the MTT assay. Both assays are described in Materials and Methods. Each dot represents the average of two measurements. r^2^ and the *p* values were calculated with GraphPad Prism 6 software.

As presented in Figure 3 there is a positive correlation between surface PS on cancer cells and their resistance to a single dose of irradiation. In addition, the data show that the higher the dose of radiation, the stronger the correlation. Since irradiation selects for cells with higher surface PS it may make tumors less sensitive to future irradiation. Interestingly, these results suggest that the amount of surface PS on clinical specimens may be used as a marker to indicate whether the tumor will be sensitive to irradiation. In addition, we hypothesized that the increase in surface PS may make the tumors more susceptible to the PS-targeting drug SapC-DOPS.

### A single dose of irradiation has modest or no effect on SapC-DOPS-induced cell death

**Figure 4:**
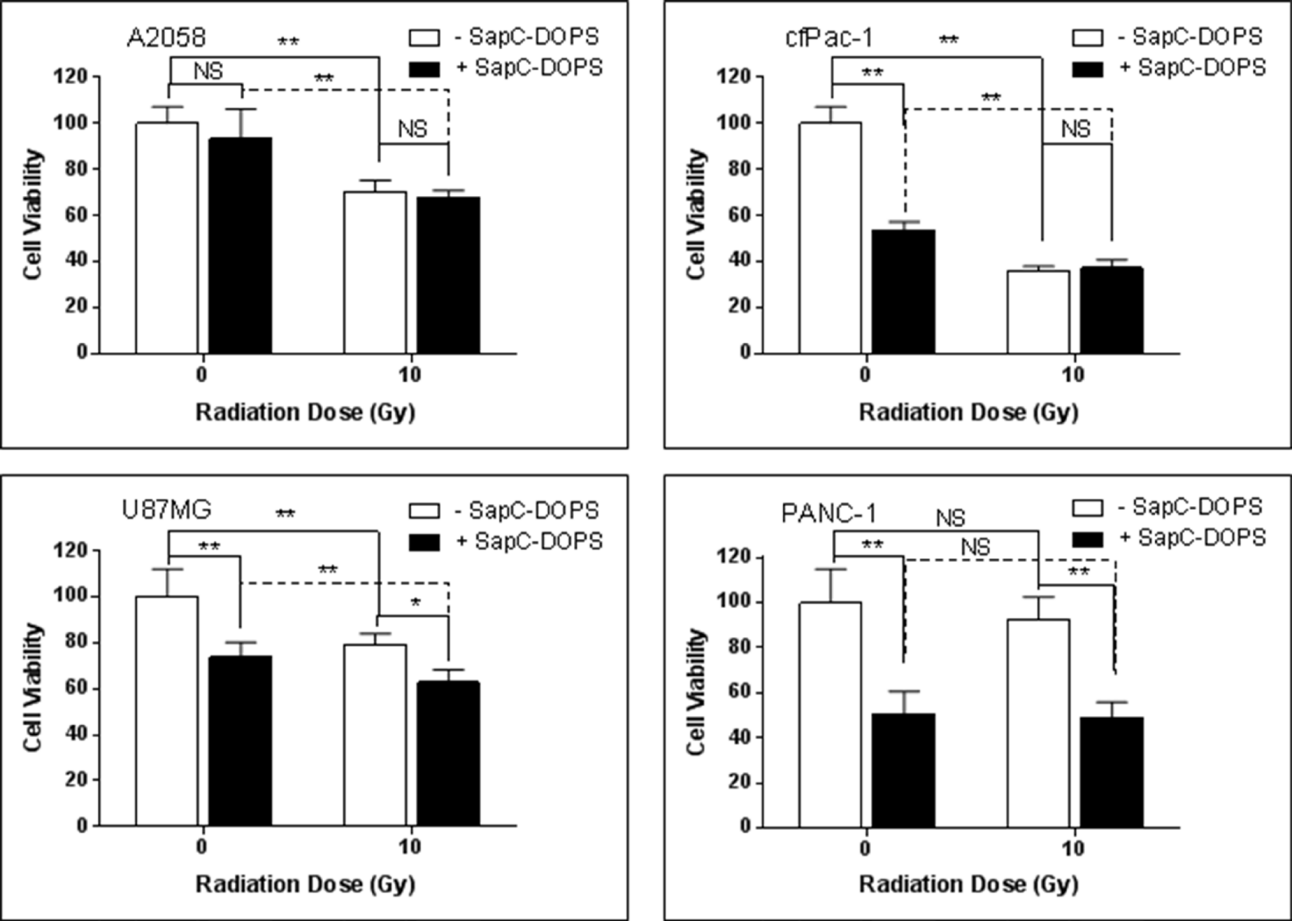
Single irradiation has modest or no effect on SapC-DOPS-induced cell death Cells were irradiated at 10 Gy and 24 hours SapC-DOPS was added. Cell death was analyzed 72 hr. later with the MTT assay. The SapC-DOPS concentration was 25 μM for A2058, cfPac-1 and PANC-1 but 40 μM for U87MG. Shown is the % of live cells compared to the control (no irradiation, no SapC-DOPS) for each cell line. ^*^p < 0.05, ^**^p < 0.01, NS = not significantly different from control.

Contrary to expectations, a single dose of 10 Gy, although it increased the proportion of cells with higher surface PS (see Figure 1), did not enhance the cell killing ability of marginally effective doses of SapC-DOPS in either A2058 or cfPac-1 cells, and only showed modest augmentation in U87MG cells (Figure 4). This may be due to the increased surface PS that occurs at the early stages of apoptosis. Since these cells are already dying, additional cell death with SapC-DOPS would not be expected. There was also no improvement of SapC-DOPS activity in PANC-1 cells since they already had a high surface PS.

### Serial irradiation of cancer cell lines makes them less sensitive to subsequent radiation and to chemotherapeutic drugs but more sensitive to SapC-DOPS

**Figure 5:**
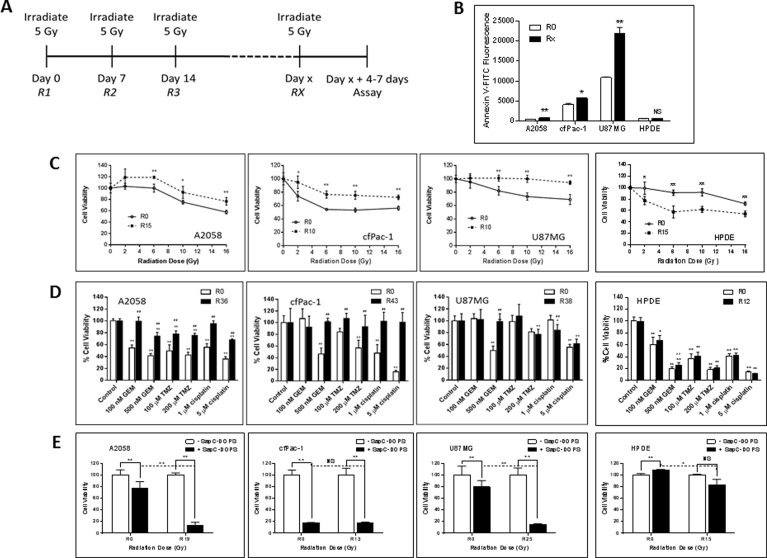
Serially irradiated cells maintain higher surface PS and are less sensitive to subsequent radiation exposure or the effects of chemotherapeutic drugs but are more sensitive to the effects of SapC-DOPS A2058, cfPac-1, U87MG and HPDE were irradiated at 5 Gy every week for various numbers of weeks (the Rx is the number of weeks the cells were irradiated. Non serially-irradiated cells are designated R0. The media was changed immediately after the irradiation and the cells were split every 3–4 days. Treatments were given 4–7 days following the last 5 Gy irradiation, when the surface PS is still elevated, (**A**) Timeline of the treatment regime. (**B**) Surface PS was determined by Annexin V-FITC staining on Day 4 following the last 5 Gy irradiation. Rx; x = 15 for A2058 and 10 for cfPac-1 and U87MG and 13 for HPDE. ^*^p < 0.05, ^**^p < 0.01 compared to R0. Shown are the mean and S.D. (**C**–**E**) Cells were serially irradiated with 5 Gy. (C) 7 days later they were exposed to the indicated doses of radiation and cell viability was determined by MTT assay was performed 72 hr. later. Shown is the % of live cells with 100% as the normalized control for each cell line. ^*^p < 0.05, ^**^p < 0.01, compared to R0. (D) 7 days after the last 5 Gy irradiation, cells were treated with different doses of gemcitabine (GEM) temozolomide (TMZ) or cisplatin and cell viability was determined by MTT assay was performed 72 hr. later. ^#^*p* < 0.05, ^##^*p* < 0.01, compared to R0 at the same dose of drug. (E) 7 days following the last 5 Gy irradiation, cells were treated with the indicated doses of SapC-DOPS (1 SapC:7 DOPS, Mol:Mol) and cell viability was determined by MTT assay was performed 72 hr. later. As in Figure 3, the SapC-DOPS concentration was 25 mM for A2058 and cfPac-1, 40 mM for U87MG and 50 mM for HPDE. Shown are the % of live cells with 100% as the normalized control for each cell line (as well as for the R0 and Rx). ^*^p < 0.05, ^**^p < 0.01, NS = not significantly different.

Since fractionated radiation therapy is a safe treatment regimen used routinely in the clinic, we performed serial irradiation of cells, weekly with 5 Gy for many weeks. During this time the cells were split every 3–4 days. The cells were analyzed 4–7 days following the final irradiation (see Figure 5A). As shown in Figure 5B, the serially irradiated cells had significantly higher surface PS than non-irradiated cells. These cells became more resistant to a single subsequent dose of radiation (Figure 5C) or to chemotherapeutic drugs, i.e., gemcitabine, temozolomide and cisplatin (Figure 5D). However, quite interestingly, serially irradiated A2058 and U87MG became more susceptible to SapC-DOPS treatment (Figure 5E). cfPac-1 was very sensitive to SapC-DOPS to begin with and the serial irradiation did not significantly enhance this.

To determine whether serial irradiation also affected normal cells we used immortalized but non-transformed human pancreatic ductal epithelial cells (HPDE). Unlike cancer cells, following serial irradiation the HPDE cells became more sensitive to radiation and remain sensitive to chemotherapeutic drugs, although the surface PS did not change. Sensitivity to a very high dose of SapC-DOPS was only slightly increased after serial irradiation.

### Combination treatment with radiation and SapC-DOPS reduces tumor growth better than either therapeutic alone

**Figure 6:**
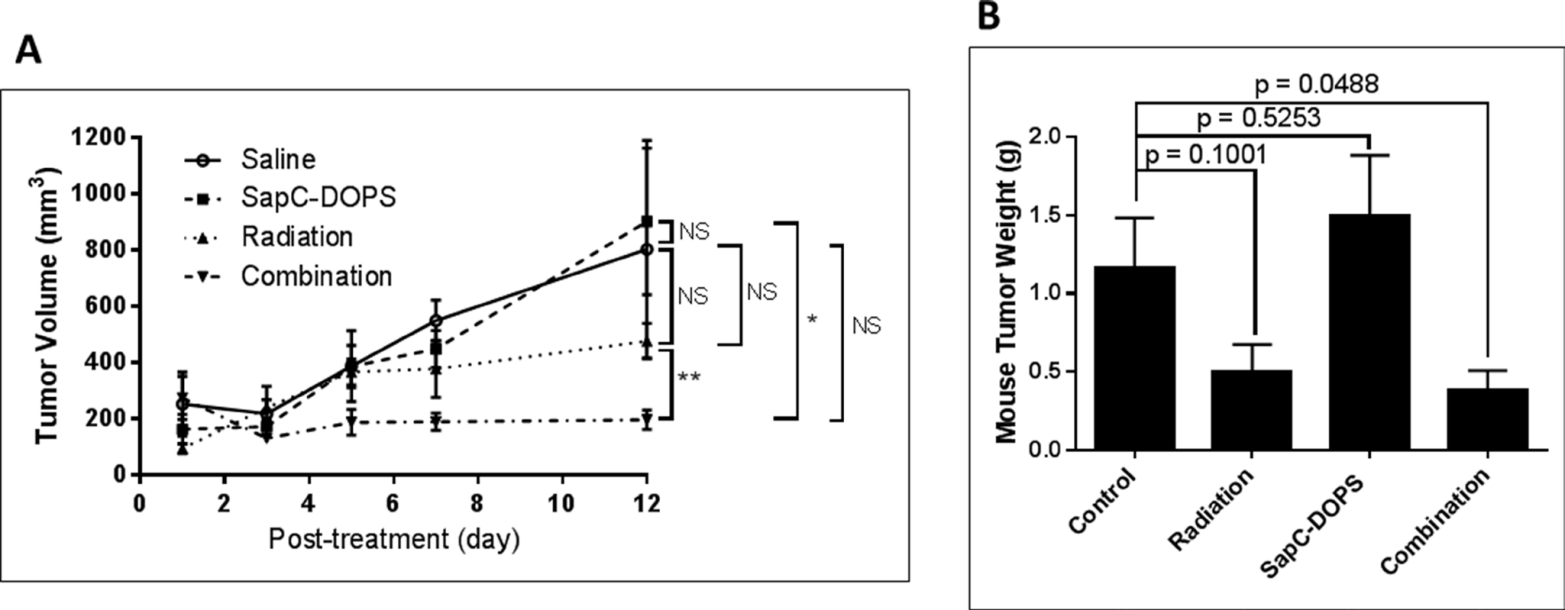
Combination of radiation and SapC-DOPS is more effective at reducing tumor size than either treatment alone Mice were injected subcutaneously with 1 × 10^6^ NCI-H460 cells. When tumors reached ~100 mm^3^, the mice were irradiated at 10 Gy as in “Materials and Methods” on days 9 and 16 following injection of the cells. SapC-DOPS (4 mg SapC/kg) was injected via the tail vein on days 11, 14, 15, 16, 17 and 18. (**A**) Tumors were measured with calipers and volumes were calculated as 0.52 × W × L2 where W = width and L = length of the tumor. ^*^p < 0.05, ^**^p < 0.01, NS = not significant. (**B**) The mice were euthanized on day 22 and the tumors were removed, rinsed with saline and weighed. *p* values from *t*-tests are shown. *n* = 4–5.

NCI-H460 cells injected into mice quickly form tumors. We selected doses of radiation (10 Gy) and SapC-DOPS (4 mg/kg) that did not significantly attenuate tumor growth by themselves. However, when used in combination, there was a significant reduction in tumor size (Figure 6).

## DISCUSSION

Radiotherapy is a common modality for a variety of cancers and may be used alone or in combination with surgery, chemotherapy, or both. The intent is that it will cure the cancer, either by eliminating the tumor, preventing metastasis or blocking recurrence. However, recent data suggest that, in some cases, the low doses given during fractionated radiotherapy may actually increase the growth and metastasis of some tumors [19, 33] and make the cancer cell more resistant to subsequent doses of radiation [33–35]. Cancer cells and their attendant blood vessels in tumors have high surface PS compared to normal cells [3, 6–8] and a positive correlation between surface PS and malignancy has been demonstrated [3, 36]. Herein we show that exposure to a single dose of irradiation induces a rapid and consistent externalization of PS in viable cancer cells with initially low to moderate surface PS both *in vitro* and *in vivo*. This increase in surface PS is both dose- and time-dependent and is on viable cells (non-PI^+^) but probably reflects the early stages of apoptosis as Z-VAD fmk inhibited the increase in surface PS. Cell lines contain a mixture of low and high surface PS populations. While most of the cells remain viable and the cells that did die may have been populations with lower surface PS (see histogram of cells in Figure 1C). Indeed, we were able to serially irradiate cells weekly at 5 Gy for over a year (data not shown). After each irradiation some of the cells died but the average PS on the remaining viable cells was increased suggesting that the lower PS cells were dying so the radiation was selecting higher PS cells. Radiation has previously been shown to increase surface PS on tumor blood vessel cells, both *in vitro* [37] and *in vivo* [21–23] and to increase plasma PS [38]. This increased surface PS enhances the effects of the PS-targeting antibody, bavituximab, for triggering the death of tumor-associated endothelial cells and thus, the tumor [39]. We did not detect an increase in surface PS on endothelial cells *in vitro* but this may have been due to the type of endothelial cells, normal HUVEC versus the previously used brain tumor-derived bEnd 3 cells.

Flippases and scramblases are Ca^2+^ and ATP-dependent aminophospholipid translocases that transport PS across the plasma membrane [40]. Flippases “flip” PS from the outside of the membrane to the inside while scramblases can transport PS in either direction. Flippases are inactivated during apoptosis while scramblases are stimulated. We have previously reported that the inherent high surface PS of cancer cells is due to reduced flippase activity probably triggered by high intracellular calcium [14]. The mechanism for the increase in surface PS in the irradiated cancer cells appears to be dependent on caspase activity as the pan-caspase inhibitor, Z-VAD fmk, completely eliminated the effect. While the caspase-dependence suggests apoptosis, the cells were PI negative at this time and didn't show significant cell death until several days later. Radiation has previously been shown to increase caspase activity [41] and caspase can cleave and inactivate flippase [42, 43], which allows PS to accumulate on the exterior of the cell. Our data, however, indicate that the increase in surface PS is not due to a reduction in flippase activity or an increase in scramblase activity (Table 1). Although not significant, scramblase activity tended to decrease which is the opposite of what we would expect for an increase in surface PS [44]. An increase in intracellular calcium can also inhibit flippase [45] but this was unchanged with irradiation. Miyato *et al*. [46] have demonstrated that PS can induce apoptosis in CHO cells using a caspase other than the typical isozymes. This caspase is inhibited by Z-VAD-fmk, but is not caspase 1, 3, 8 or 9. It is possible that this unidentified caspase induces externalization of PS by a phospholipid translocase-independent mechanism. Finally, total PS, in comparison to other membrane phospholipids, although constitutively increased in cancer cells with high surface PS [14], was unaffected by radiation (Table 1).

As mentioned, surface PS is associated with greater malignancy [3, 36] and we show here, an inverse relationship between cancer cell surface PS and sensitivity to radiation (Figure 4). On the other hand, our previous data indicate that cancer cells with elevated extracellular PS are more susceptible to SapC-DOPS [6, 15, 29]. Therefore, we hypothesized that SapC-DOPS could kill cells that are resistant to radiation and that radiation may actually enhance the performance of SapC-DOPS.

The protein/lipid nanovesicle, SapC-DOPS shows a robust microscopic tumor-seeking activity in preclinical cancer models via a PS-mediated targeting pathway [15, 24, 29]. However, unlike most standard therapies, the nanovesicles are cytotoxic to cancer cells with diverse genetic profiles without induction of acquired resistance [6, 11]. We have previously demonstrated that SapC-DOPS synergizes with the chemotherapeutic drugs, temozolomide [47] and gemcitabine (unpublished data). However, a single dose of radiation (10 Gy) provided little or no enhancement of SapC-DOPS activity. We, therefore, examined the effects of serially irradiating the cells at 5 Gy weekly as described by Lee *et al*. [35]. Their data as well as ours (Figure 5C) and others [34] indicate that cancer cells become more resistant to radiation with repeated exposure to low dose radiation which is commonly used in fractionated treatment [48]. This increased resistance is accompanied by an increase in surface PS. Interestingly, serially irradiating cells also makes the cells more resistant to chemotherapeutic drugs. Although combining radiation with chemotherapy is standard practice [17, 18, 49], there may be diminishing returns with multiple cycles of radiation treatment. Of course our *in vitro* data may not reflect clinical outcomes in patients.

Importantly, serial irradiation significantly improves SapC-DOPS efficacy depending on the cell line, with some cell lines responding much stronger to the sequential therapy compared to others – the one cell line that did not show the enhancement also had the least significant increase in PS. The surface PS of untreated U87MG cells is higher than A2058 or cfPac-1 even after the latter cell lines have been serially irradiated but U87MG is less sensitive to SapC-DOPS. U87MG is a glioblastoma cell line and we have previously shown that these tumor cells undergo lysosomal destabilization in response to SapC-DOPS instead of apoptosis as is seen in most other cancer cells [50]. Indeed our previous studies has shown that U87MG are relatively resistant to SapC-DOPS compared to other cells with comparable surface PS so factors other than surface PS can apparently influence SapC-DOPS-induced cell killing. The saposin C of SapC-DOPS catabolizes sphingomyelin to ceramide [15], which is toxic to cells. Radiation also transiently increases ceramide [51, 52]. Accordingly, multiple doses of radiation may increase ceramide above that obtained with a single dose and enough to enhance the SapC-DOPS effect. Sphingomyelin plays a role in the microenvironment of tumor cells and accelerates angiogenesis [53] so the breakdown of sphingomyelin may also provide a mechanism for this combination therapy.

Our data suggest that radiation targets low surface PS cells while SapC-DOPS works better on higher surface PS cells so a combination of these modalities would result in enhanced tumor cell death (see Schema 1) as we observed in cancer cells (Figure 5) and in mouse tumors (Figure 6). The improved efficacy of SapC-DOPS with fractionated irradiation may have significant benefits in the clinic as SapC-DOPS, which has shown no significant side effects in mice [6, 11, 24], or so far in humans (27), may allow for a lower effective dose of radiation with fewer normal tissue toxicities.

**Schema 1:**
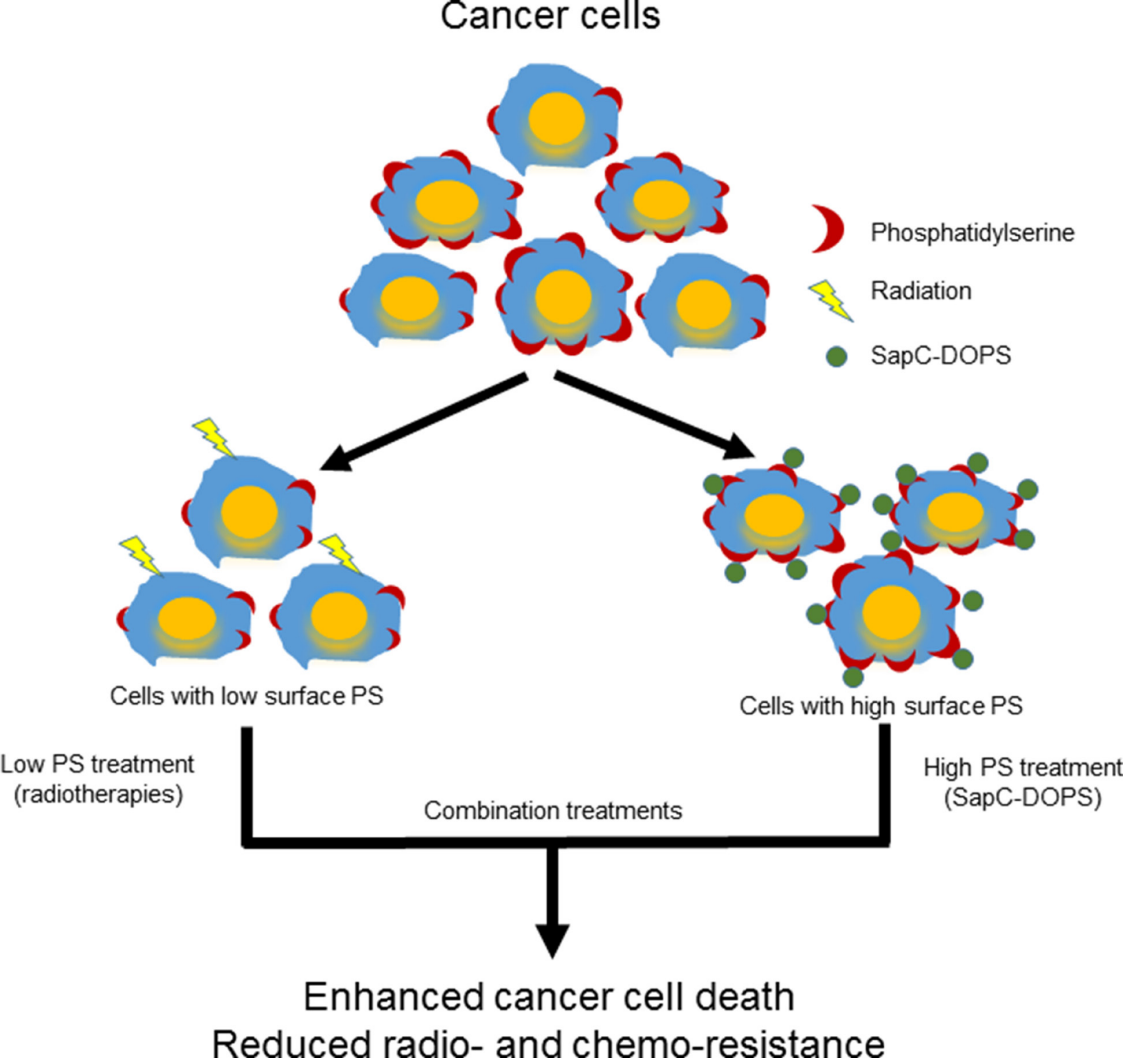
Phosphatidylserine-selected therapies of SapC-DOPS nanovesicles and radiation to enhance cancer cell death

## MATERIALS AND METHODS

### Cell lines

Human cancer cell lines, A2058, AsPC-1, SKNSH, MiaPaCa-2, cfPac-1, NCI-H460, H1915, H1299, U87MG and U373 and the normal pancreatic cell line (HPDE) were obtained from ATCC (Manassas, VA, USA). MDA-MB-231-luc- were obtained from Caliper Life Sciences (Mountain View, CA, USA). HUVEC were from Lonza (Basel, Switzerland). HPDE, A2058, AsPC-1, MiaPaCa-2, cfPac-1, U87MG, Gli36 and U373 were cultured in DMEM (Fisher Scientific, Pittsburgh, PA, USA). H460, H1299 and H1915 were cultured in RPMI 1640 (Fisher Scientific). MDA-MB-231- Luc and SKNSH were cultured in AMEM (Invitrogen, Carlsbad, CA, USA). The above cell lines except HUVEC, were cultured in their respective media supplemented with 10% FBS and 1% Penicillin/Streptomycin. HUVEC were grown in EGM-2 media (Lonza).

### Flow cytometric analyses of annexin V binding

Unless otherwise stated, cells at 60–80% confluency were irradiated and then, after the appropriate time, were trypsinized, resuspended in complete medium, spun down, and washed once with PBS then resuspended in annexin V staining buffer. Cells (1 × 10^5^) were incubated at room temperature in the dark for 15 minutes in a final volume of 100 μl containing 10 μl Annexin V-FITC (Invitrogen, Carlsbad, CA, USA) and 2 μg/ml propidium iodide (PI; BD-Pharmingen, Eugene, OR, USA). Annexin V-FITC binding was measured by flow cytometry after adding 400 μl of annexin V binding buffer, using a BD Fortessa. Data was analyzed with BD FACS Diva. For quantifying the annexin V-FITC signal from living cells, PI positive dead cells were gated out and the annexin V-FITC signal was obtained from PI negative forward scattered cells.

### Cell viability assay

Cells were seeded in flat bottom 96 well plates (Falcon, Becton Dickson Labware, Franklin Lakes, NJ, USA) at 2000–12000 cells/per well (depending on the growth rate of the cell line). The number of cells seeded was adjusted to give an optical density of 0.6 to 1.2 (linear range of the assay) at the time of testing. The next day the cells were treated and 72 hr. later were analyzed for cell viability with the Cell Proliferation Kit I (MTT; Roche Diagnostics, Mannheim, Germany).

### Flippase and scramblase assays

Flippase was measured as previously described [14]. Briefly, cells were resuspended in 3ml of flippase assay buffer and NBD-PS (Avanti Polar Lipids) was added to a final concentration of 3 μM. Cells with NBD-PS were divided into aliquots and incubated for 0, 1, 5, 15, 30 or 45 minutes. After each incubation time, half of the cell suspension was separated for non-extracted sample and kept on ice. The remaining half was spun down to remove non inserted NBD-PS and subjected to BSA extraction of NBD-PS from the outer leaflet by adding 3% fatty acid free BSA (MP Biomedicals) in flippase assay buffer. After incubation on ice for 20 minutes, freshly prepared sodium dithionite (Sigma) was added to a final concentration of 10 mM and incubated a further 10 minutes, to reduce the NBD lipids in the outer cell surface. The cells were spun down and resuspended in flippase assay buffer containing 0.25% BSA and 2 μg/ml PI. NBD-PS signal from unextracted and extracted samples was measured by flow cytometry from living cells after exclusion of PI-positive dead cells, using a BD Fortessa. Non-extractable NBD-PS in the BSA and sodium dithionite treated sample was presented as the percentage of total amount in the control unextracted sample. Scramblase was measured in the same way but with NBD-PC (Avanti Polar Lipids).

### Measurement of intracellular calcium

Intracellular calcium was measured by using the calcium binding dye Fluo-3 AM (Invitrogen). Cells (1 × 10^5^) were loaded with 5 μM final concentration of Fluo-3 AM and incubated for 30 min at 37°C. Cells were washed twice with DMEM, and resuspended in DMEM and incubated for a further 30 minutes at 37°C. The cells were washed twice with PBS +2% FBS and resuspended in 400 ml of this. Two μg/ml PI was added and the Fluo-3 AM signal was measured by flow cytometry after exclusion of PI positive dead cells, using a BD Fortessa cytometer. Data was analyzed using BD FACS Diva software.

### Thin layer chromatography (TLC) and quantification of total phosphatidylserine

Total cellular lipids were extracted by chloroform/methanol extraction. TLC was performed as previously described [14]. Lipids were loaded onto a TLC plate based on protein quantification. Brain PS (Avanti Polar Lipids) and sphingomyelin (Matreya LLC) were run as molecular standards. Bands corresponding to PS were scraped and subjected to phosphorus extraction by acidic digestion. The liberated phosphorus was estimated by allowing a complex formation with ammonium molybdate (Sigma) and malachite green (Sigma) and by measuring the absorption at 660 nm [54]. Phosphorus was quantified using a standard curve obtained from phosphorus liberated from known concentrations of brain PS run on TLC plate. Cellular PS was expressed as the ratio of phosphorus obtained from PS and phosphorus obtained from total phospholipids. Additionally, PS was estimated by acquiring TLC band intensities of PS and sphingomyelin, using Image Studio Lite software; variations in PS are shown as a ratio of PS to sphingomyelin bands.

### Tumor growth in mice

All animal studies were approved by the Institutional Animal Care and Use Committee of the University of Cincinnati (Protocol Number: 11-05-05-02). Female nude/nude athymic mice (Taconic Farms, Germantown, NY, USA) weighting approximately 20–25 g were injected with cancer cells (1–2 × 10^6^ cells in 0.2 ml PBS) subcutaneously in the right shoulder or flank. When the tumors were approximately 400 mm^3^, they were irradiated with targeted radiation as below. After 48 hr. the tumors were removed, dissected, and dissociated from a 30 mg portion with a kit from 101Bio (Palo Alto, CA, USA). The harvested cells were stained with Annexin V-FITC and PI and subjected to flow cytometry. In other experiments, mice were treated with SapC-DOPS (prepared as in Chu *et al*. [6]) following the irradiation and the tumor sizes were measured for up to 15 days.

To examine radiation and SapC-DOPS on tumor growth, mice were injected with 1 × 10^6^ NCI-H460 cells. When the tumors reached 100 mm^3^ the mice were treated with 10 Gy irradiation and/or 4 mg/kg SapC-DOPS. At the end of the experiment the tumors were removed and weighed.

### Irradiation

Cells were grown to 60–80% confluency in 60 mm dishes then exposed to (^137^Cs) irradiation with a GammaCell 40 Exactor (Nordion International) at doses of 0, 2, 6, 10 or 16 Gy. The media was changed and the cells were processed for surface PS determination at the indicated times. Mice were irradiated at 10 Gy with an Xstrahl D3100 superficial systems (at 100 kV) with 2 cm cone and projected 1 cm deep.

### Statistical analyses

Differences between treatment groups were determined by t-test, Pearson correlations were obtained with GraphPad Prism and data are presented as mean ± standard deviation.

## Abbreviations

PS: Phosphatidylserine
SapC: saposin C
DOPS: dioleoylphosphatidylserine
Ca^2+^_i_: intracellular calcium
